# Mollusks of Candomblé: symbolic and ritualistic importance

**DOI:** 10.1186/1746-4269-8-10

**Published:** 2012-03-15

**Authors:** Nivaldo A Léo Neto, Robert A Voeks, Thelma LP Dias, Rômulo RN Alves

**Affiliations:** 1 Programa de Pós-Graduação em Ciências Biológicas (Zoologia), Universidade Federal da Paraíba, João Pessoa, PB, Brasil; 2 Geography Department, California State University, Fullerton, 800 N. State College Blvd, Fullerton, CA 92834, USA; 3 Departamento de Biologia, Universidade Estadual da Paraíba, Av. das Baraúnas, 351/Campus Universitário, Bodocongó, Campina Grande, PB 58109-753, Brasil

**Keywords:** Ethnozoology, symbolic systems, Use of mollusks, Afro-Brazilian religion

## Abstract

Human societies utilize mollusks for myriad material and spiritual ends. An example of their use in a religious context is found in Brazil's African-derived belief systems. Candomblé, an Afro-Brazilian religion introduced during the 18^th^-19^th ^centuries by enslaved Yoruba, includes various magical and liturgical uses of mollusks. This work inventoried the species utilized by adherents and to analyzed their symbolic and magical context. Data were obtained from Candomblé temples in two cities in the northeast of Brazil-Caruaru, in the state of Pernambuco, and Campina Grande, in the state of Paraíba. Questionnaires administered to eleven adepts revealed that at least nineteen mollusk species are being used. Shells from *Monetaria moneta, M. annulus *and *Erosaria caputserpentis *were cited by all of the interviewees. Three uses stood out: divination (*jogo de búzios*); utilization as ritual objects; and employment as sacrificial offerings (*Igbin *or *Boi-de-Oxalá*). The *jogo de búzios *(shell toss), employed in West Africa, Brazil and Cuba, is of fundamental importance to the cult, representing the means by which the faithful enter in contact with the divinities (*Orixás*) and consult people's futures (*Odu*). The utilization of mollusks in Candomblé is strongly influenced by ancient Yoruba myths (*Itãs*) which, having survived enslavement and generations of captive labor, continue to guide the lives of Brazil's African Diaspora.

## Background

Human societies utilize aquatic biological resources in many ways. Marine invertebrates and fish are used as ornamentals, food, medicine, and even in magico-religious practice [1-15]. Interactions between humans and animals go well beyond simple ecological and functional relations and, since antiquity, cultures have attributed magical and religious meaning to wild and domesticated animals [7,8,16-19]. Particularly in pre-scientific societies, according to Marques [5], the imaginative that is present in the daily routines of people has encouraged mystical and religious relations with the environment.

In Brazil, the use of animals in magic-religious areas is widely reported but infrequently investigated [8,20-24]. Although a diverse array of wild animals and animal parts are offered for sale for religious purposes throughout the country in the open markets and shops, especially to serve the Afro-Brazilian community [7,8,15,23,25,26], few studies have analyzed their use, especially in terms of a conservation framework. As Costa-Neto [27] notes, utilization of wild animal species in a magic-religious context has been mostly neglected by biological scientists, who often find their respect for traditional and indigenous uses of nature in conflict with their developed world, environmental ideology.

The use of mollusks is probably as old as humanity. In pre-history, mollusks represented an important source of food, ornamentals, and tool materials [28-31], and these traditional uses are richly documented in the archeological record [32-34]. Mollusks continue to be an important source of food, providing an essential source of protein in the coastal communities [35-38]. But in addition to their consumptive value, their shells have long been utilized for magical-religious purposes. During the European Middle Ages, for example, the shell of the pilgrim (*Pecten jacobaeus*; a bivalve mollusk) became a religious emblem of Saint James. The shells of the *P. jacobaeus *can be seen in sanctuaries or as church decorations, such as those in Santiago (Spain) and Porec (Croatia). And in island societies of the Indian Ocean it is used it as a symbol of love and fertility [39].

As a largely immigrant landscape, Brazil's cultural relationship with nature is guided as much by Old World as with New World traditions. In addition to Amerindian biocultural relations, Portuguese, Italian, German, Japanese, and especially Africans introduced and intermixed their beliefs and practices. Particularly in the northeast of Brazil, where some two million enslaved Africans arrived over the course of three centuries, people-animal relations were significantly shaped by the Yoruba of present day Nigeria and Benin. With a rich religious tradition and a profound knowledge of the magical and medical properties of plants and animals, the Yoruba introduced many of their healing practices and cosmological beliefs to Brazil [40]. Over time, as these beliefs and traditions blended with those of the Catholic faith, a syncretic religion known now as Candomblé (and many variants) took hold. Candomblé priests and priestesses serve the greater Afro-Brazilian community as priests, *curandeiros *(healers), and practitioners of the occult arts. These often include plants and animals in ceremonial obligations (sacrifices) to one or another of the pantheon of African deities-the *orixas *[7,8,15]. Ritual activities, including use of wild and domesticated biota, are often guided by a complicated set of ancient African oral myths-the *odu *[41-45].

An coterie of animals are employed in Candomble ceremonies, native and exotic, terrestrial and aquatic. These include goats, chickens, doves, guinea fowl, snakes, dolphin, fish, and many others [7,8,15,23,25,26]. Among these, mollusks hold a prominent role [7,8,15,46-54]. From anthropological perspective, the importance of gastropods to Candomblé has been briefly noted by other researchers [42,43,51,55]. The present work is the first ethnozoological study that examines the role and general significance of mollusk's species to Candomblé practice. The objectives of the following research are: (1) to identify species of mollusks that are used by Candomblé priests, and (2) to analyze the roles and magic-religious symbolism of these animals to the adepts of the religion.

## Materials and methods

Data were collected in the cities of Caruaru, in the state of Pernambuco, and Campina Grande, state of Paraíba, in the northeast of Brazil. The city of Caruaru (8°17'00"S; 35°58'34"W) is situated 132 km from the state's capital, Recife, occupies an area of 10,117 km^2^, and has a population of approximately 289,000 inhabitants. The city of Campina Grande (7°13'11"S; 35°52'31"W) is located 112.9 km from the state's capital, João Pessoa, and has a population of roughly 371,000 people [56].

Field data were collected through semi-directive [57], and non-directive interviews and informal discussions [58-60] with 11 priests and priestesses from the Candomblé. The study was carried out between the months of August 2007 and June 2008. Approval for the study was obtained from the Ethics committee of Universidade Estadual da Paraíba. We gained access to the terreiros through holy sons (filhos-de-santo) known to the first author. This previous relationship facilitated trust, enabling him to conduct interviews with priests and priestesses. Additional interviewees were chosen by using the snowball sampling technique. Some attempts to interview Candomblé priests and priestesses were unsuccessful due to inaccurate information regarding their location, and some interviewees provided little information because they were reluctant to answer questions. As pointed by Leo Neto et al. [56] rituals are often carried out in secrecy because the predominantly Christian society is antagonistic to this religion, which makes it challenging to gain access and information regarding these practices.

Species were collected and identified to the lowest taxonomic level possible utilizing the relevant literature [61-63]. Some species were identified during interviews and, when necessary, photographed for later analysis. Binomials were determined using the database Malacolog, Version 4.1.0 [64].

## Results and discussion

A total of 19 mollusk species possessing liturgical and symbolic ends were identified in the present study. The most cited species were: *Monetaria annulus *(*n *= 10 citations), *M. moneta *(*n *= 10), *Erosaria caputserpentis *(*n *= 9) and *Achatina fulica *(*n *= 6). The families with the greatest number of registered species were: Cypraeidae (*n *= 6) and Ranellidae (*n *= 2); the other families were represented by one species each (Table 1). Silva [55], who carried out a similar study in the Brazilian cities of Recife and Olinda (Pernambuco), registered a total of 11 species utilized in magico-religious ceremonies. Of these, only the genus *Cypraea *sp. and the species *Cassis tuberosa*, *Eustrombus goliath *and *Charonia variegate*, were also encountered and used for similar purposes in the Candomblé *terreiros *(the physical space where the religious rituals are performed) visited in this study.

**Table 1:** Mollusks used in Candomble temples, including uses, symbolism, and liturgy.

Family/Species	Name(s)	Geographic range	Uses	Number of mentions	
				CG	CA
Achatinidae					
*Achatina fulica *(Ferussac, 1821)	*Ibi*, *Igbin*, Boi-de-Oxalá	Native to East Africa, introduced into other countries, including Brazil	Sacrificial offerings to Oxalá	--	6
Cassidae					
*Cassis tuberosa *(Linnaeus, 1758)	---	North Carolina, Caribbean and Brazil	Used ornamentally on the shell game table used by the temple priest during consultation; ornamental use for Oxumarê's altar	--	2
Cerithiidae		-			
*Cerithium eburneum *Bruguière, 1792	---	Southeastern Florida and West Indies to Brazil	Present on the divination table of a Yoruba nation babalorixá	--	1
Cypraeidae					
*Monetaria annulus *(Linnaeus, 1758)	Búzio	Indo-Pacific	Used in shell game; ornaments used in altars and liturgical objects	2	8
*Erosaria caputserpentis *(Linnaeus, 1758)	Búzio-africano	Indo-Pacific	Used in shell game; ornaments used in altars and liturgical objects	1	8
*Monetaria moneta *Linnaeus, 1758	Búzio	Indo-Pacific	Used in shell game; ornaments used in altars and liturgical objects	2	8
*Cypraea tigris *Linnaeus, 1758	---	Indo-Pacific	Symbolizes the "*odus*"(sacred oral text) for a Yoruba nation temple priest	--	1
*Macrocypraea zebra *(Linnaeus, 1758)	---	North Carolina to Yucatan, West Indies to Brazil	Present on the divination table of a Yoruba nation temple priest	--	1
*Luria cinerea *(Gmelin, 1791	---	North Carolina to Yucatan, West Indies to Brazil	Present on the divination table of a Yoruba nation temple priest	--	1
Muricidae					
*Stramonita rustica (Lamarck, 1822)*	---	Florida to Brazil	Present on the divination table of a Yoruba nation temple priest	--	1
Neritidae					
*Nerita fulgurans*	---	Southeastern United States to Brazil	Present on the divination table of a Yoruba nation temple priest	--	1
Ostreidae					
*Crassostrea rhizophorae *(Guilding, 1828)	Ostra	Caribbean to Brazil	Offering to Iemanjá	1	---
Pectinidae					
*Euvola ziczac *(Linnaeus, 1758)	---	North Carolina to Brazil	Ornament for an Oxum liturgical object	--	1
Ranellidae					
*Cymatium raderi *D'Attilio & Myers, 1984	---	Honduras, Tobago Is., and Brazil	Ornament for Oxumarê's altar	--	1
*Charonia variegata *(Lamarck, 1816)	---	North Carolina to Brazil, Cape Verde, Ascension and Sta. Helena Island	Table ornament where a temple priest consults using the shell game	--	1
Spondylidae					
*Spondylus americanus *Hermann, 1781	---	North Carolina to Brazil	Altar ornament for Yemanjá	--	1
Strombidae					
*Eustrombus goliath *(Schröter, 1805)	---	Northeastern and Southeastern Brazil	Table ornament where a temple priest consults using the shell game; altar ornament for Yemanjá	--	1
Turbinidae					
*Lithopoma tectum *(Lightfoot, 1786)	---	Campeche to Brazil	Present on the divination table of a Yoruba nation temple priest	--	1
Veneridae					
*Anomalocardia brasiliana *(Gmelin, 1791)	---	West Indies to Brazil	Present on the divination table of a Yoruba nation temple priest	--	1

The mollusk species recorded in this study come from many regions of the world. The majority was harvested from the Atlantic Ocean, but quite a number of species (n = 5) were imported from Indo-Pacific countries. The presence of exotic species in the NE Brazilian trade suggests that Brazil is an important market for shell trade [3]. The use of exotic cowries such as *Monetaria annulus *and *M. moneta *indicating the importance of these species in regional and global trading.

In addition to religious uses, some of the species identified in this work are utilized for various purposes in Brazil. For example, Alves *et al.*[11] and Dias *et al.*[3,12] registered species of *Cassis tuberosa *and *Eustrombus goliath *being used by craftsmen for the production of pieces of art. In a bibliographic revision of animals used in traditional Brazilian medicine, Alves *et al.*[14] and Alves and Dias [2] reported that *C. tuberosa*, *Anomalocardia brasiliana *and *Crassostrea rhizophorae *are utilized in the treatment of illnesses, including asthma, stomach aches, and osteoporosis. As Alves and Rosa [65] point out, the multiple of uses of mollusks--artisanal, medicinal, and magic-religious--should be investigated in terms of possible environmental impacts on the species.

In the present study, three sets of religious traditions uses stood out: divination (*jogo de búzios*); utilization as ritual and decorative objects; and employment as sacrificial offerings (*Igbin *or *Boi-de-Oxalá*).

## Divination: *Jogo de Búzios*

The *jogo de búzios *(literally shell game), referred by some authors as *dilogun *[66], *dologun *[43] or *erindilogun *[51], assumes a fundamentally important role in the religious context of Candomblé. The *búzios *constitute the principal means of exchange and communication between humans and the pantheon of African deities, the *orixás*. Among these deities, *orixá *Exu is most integral to the success of the *búzios*. As the owner of streets and crossroads, the arteries of communication, Exu symbolically directs traffic between the parallel worlds of *Aiyê *(the realm of the mortals) and *Orum *(the realm of spirits). During divination via the *jogo de búzios*, Exu transmits the messages of the divinities to the *terreiro *priest or priestess (*pai/mãe de santo*) who in turn translates them for the client or adept [45].

The *búzios *are fundamental to a number of *terreiro *activities, first among these being divining the future for a client or adherent. For non-adherents, this can entail any of the major and minor life events, for example, "will my business venture be successful?", "will my daughter's health improve?", "will my wife return to me?". In many instances, the money generated by this service represents a primary means of supporting the priest or priestess. Nonetheless, the religious importance of *jogo de búzios *goes beyond the divinatory practices, the "*olhar o futuro*" (eye of the future) of the consultant. Through the *búzios*, the *orixás *are able to converse with the faithful. By means of these conversations, believers are able to learn: (1) the principal *orixá(s) *that govern the *ori *(head) of the individual. In Candomblé, each adherent is associated with, and in many respects governed, by one or two *orixás*; (2) the appropriate sacrifices and other offerings that should be made to the owners of the person's head; and (3) whether there is anything happening in a ceremony (such as *Orô*, or sacrificial cerimony) that is pleasing or displeasing the relevant deities. In Candomblé, nearly all important actions are predicated on the result of consultation by means of the *búzios*. One priest expressed the function of these mollusks by the following:

The function of the *búzios *in Candomblé is the same function in medicine that would use an exam or an ultra-sound. For everything you consult the búzios. The doctor asks an exam for everything, he will consult everything, to see how is the disease, and what it is and what it isn't (Pai J. de Ogum, Caruaru, terreiro of the Keto Nation).

Three species of mollusks were identified that were used in the *jogo de búzios*: *Monetaria annulus, M. moneta *and *Erosaria caputserpentis*. The species known as the African shell (*E. caputserpentis*), according to some of those interviewed (*n *= 5), has a greater significance in the *jogo de búzios *because of its lexical designation as "African", that is, the "land of the ancestors." This is a widespread phenomenon in Afro-Brazilian religions. Given the monumental barriers to importing liturgical animals and plants during the slave trade, those African species that did arrive or were already present--wild or domestic, animal or plant--have attained nearly sacred status [44]. To other *pais *and *mães de santo*, the shells of the species *M. annulus *or *M. moneta *are preferred, because the symbolism of their white color. According to Santos [51], the white represents the generic existence not only of *Aiyê *(the material world) but also *Orum *(the spiritual world), for this reason, considered one of the three elements that participate in the formation of everything that exists and simultaneously representing also the passage, or rather, the transformation from one level of existence to another.

The "Jogo de Búzios" is composed of 16 shells and only the priests can consult *Ifá *through these shells. Bastide and Queiroz [42], analyzing the Candomblé of Bahia, reported that some priests utilized 32 shells. In the present study, it has not been observed the use of 32 shells. The shells must go through a process of preparation before being tossed. They must go through a bath of *Amassi*, elaborated from leaves and herbs specified by the *orixá *of the adept, they also must go through a blood bath, in Yoruba called *Ejé*. That is due to the fact that in order to become sacred and vehicles of the divine word of the *orixás *to men, the búzios shells must receive *Axé*. According to Santos [51], the *Axé *would be the force that ensures the dynamic existence, allowing for certain future happenings, and without it existence would be paralyzed, deprived of the possibilities of realization. Being a force, it would, therefore, be transmittable and conduced by the material and symbolic means. Santos [51] demonstrates that "all objects, being or place is only sanctified through the acquisition of *Axé*, being that the materials of the *terreiro*, just as its initiated adepts, must receive this energy, accumulate, maintain, and develop it. According with Prandi [67], to become sacred depends on sacrifice. The plants and herbs, in this case, are considered to be "vegetable blood" in the process of making the owner sacred. According to Merrell [68], the power of Axé is contained within and transmitted by elements in the vegetal, animal, and mineral realms that are grouped in three categories: (1) "red blood" from the animal realm, the vegetal realm (copper), (2) "white blood" from the animal realm (semen, saliva), vegetal real (sap), and mineral realm (lime), and (3) "black blood" from the animal realm (ashes), vegetal realm (dark juice from certain fruits and vegetables), and mineral realm (charcoal, iron). Axé allows one's to fulfill themselves through one's maintaining a proper balance with one's animal, vegetal, and mineral environment [45,69].

The shells must also be "open." The opposite side of the natural opening of the shells is sanded open, leaving the columella (the central anatomical feature of a coiled gastropod shell) exposed.

The priest, with 16 shells in hand, using inaudible words, throws the shells counting those that fall open (with the columella facing up) and those that fall closed (with the natural opening facing up). According to *Pai *F. de Logun-Edé, *babalorixá *of the "Ketu Nation" "*the búzio toss is a mathematical study*". Beginning with the counting of the positions in which the shells fall, the sacerdotal will read that *Odu*. The *odu *can be characterized with a type of destiny of the person that consults the oracle. Each *odu *is specific and accompanied by a history, a myth of which its symbolic base interpreted by the priest will advice in a way to help the consultant in his/her life. Ribeiro [70], studying the Afro-Brazilian cults in Recife, describes the various types of *odus*, with a brief symbolic explanation of each. Bastide and Queiroz [42] also provides a list with names of *odus *mentioned by the priests in Bahia.

If there is any doubt of *odu*, the priest resorts to a more simple practice, with only 4 shells. These shells, being asked simple and objective questions, will answer positively or negatively to the questions that were asked. From this, according to one of the interviewed priests, we have the following probabilities:

• All búzios fall "open" = yes

• All búzios fall "closed" = no

• Two "open" and two "closed" = there is a necessity to ask two more times.

• One "closed" and three "open," or vice-versa = the question was not understood. Rethink the question and state it with more conviction.

**Figure 1:**
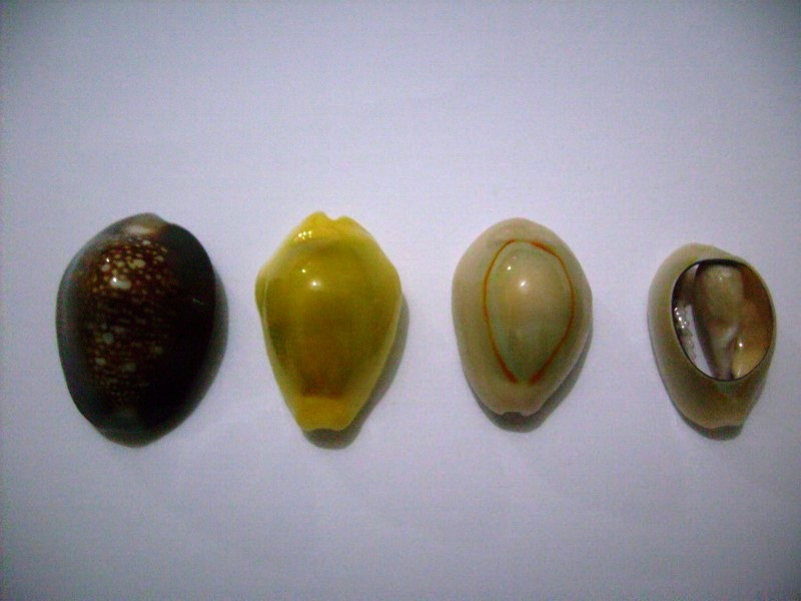
**The species of búzios found in the visited terreiros**. From left to right: *Erosaria caputserpentis*, *Monetaria moneta *and *M. annulus*. The fourth shell (*M. annulus*) is sanded, leaving the columella exposed.

**Figure 2:**
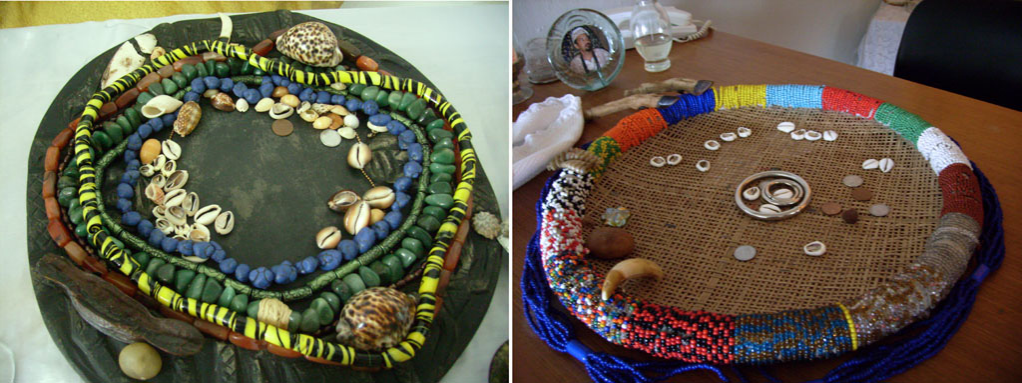
**"Strainers" where búzios shells are tossed for the divination practices**. On the left molluscs species *Cypraea tigris *(Larger shells) and Smaller (*Macrocypraea zebra*, *Erosaria caputserpentis*, *Luria cinerea*, *Lithopoma tectum *and *Cerithium eburneum*). On the right, around the edges of the strainer it can be seen two deer feet (*Mazama americana*) representing Odé, a rattle snake rattle (*Caudisona durissa*), representing Oxumarê, and the tiger tooth (Felidae) being used just for decoration without any mystical sense. The same is also decorated with necklaces representing the 16 orixás cultivated in Brazil.

The búzios are normally thrown onto a type of decorated strainer with symbolic objects (Figures 1 and 2), like parts of animals, stones, guides (type of necklace) with the specific colors of the *orixá *owner of the *ori *of the priest, among others. This strainer is known as the "*Ifá *board."

Assuming its role of communication between men and the deities, the *búzios *assume a fundamental importance in Afro-Brazilian cults. The actual role cited by some priests as the "hand-of-*Ifá" *is only known to people that possess certain time of initiation. Nonetheless, according to Ribeiro [70], the job of the "hand-of-*Ifá," *would only be granted to priests that are dedicated exclusively to the cult of *Ifá*, having as a divinatory practice the *opele*, a type of cord made up of dendê (*Elaeis guineensis*, Jacq) nuts. According the cited author, these priests are designated as Babalaô, which practically do not exist in Brazil.

According to Eliade [71], the sacred symbolic value of shells and the pearls, became, little by little, profane, given the economic value that it was given. We can make an analogy in the case of Candomblé. In formal conversations, the priests demonstrate great preoccupation around the reputation of the religion, given that people commonly see the place where "satanic cults" are carried out, as well as a means by which adepts may collect large sums of money. This erroneous vision that an uninformed population possesses, reflects on the perception that Candomblé is nothing more than a "money factory." As a result, people that do not pass the process of the necessary initiation inside these religions, think they have the right to carry out consultations through the shell toss with an extremely commercial goal, resulting in an "erroneous vision" to the Afro-Brazilian world on the part of the majority of the Christian population. In this way, the Afro-Brazilian culture dissolves into a capital world where the role of money is exalted, instead of the knowledge and zeal of the religious culture.

## Magical-religious decoration

**Figure 3:**
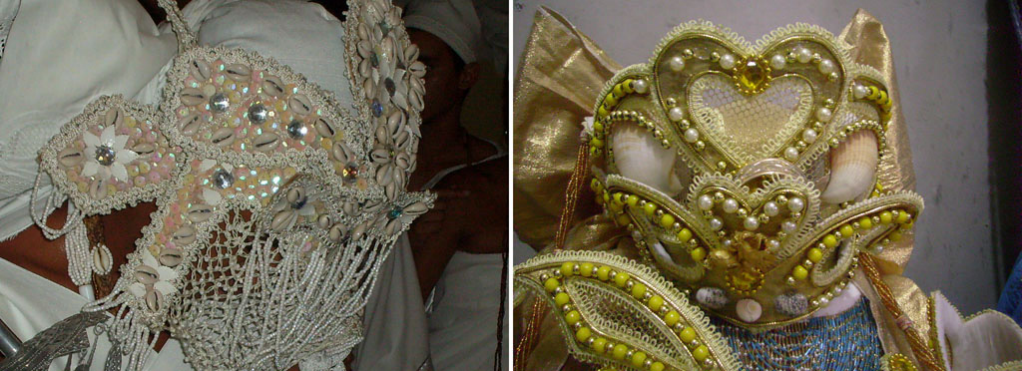
**Masks used by the *filhos-de-santo *when they incorporate their *orixás***. In the left: Details of the mask of the Oxalá-Oxalufã ornamented with the *búzios *of the species *Monetaria annulus *in the *terreiro *of the "Keto Nation." In the right: the mask of Oxum in the *terreiro *of the "Yoruba Nation" in Caruaru (PE) ornamented with shells of species *Euvola ziczac *and *Nerita *sp.

Of the 19 species registered in this work, 9 are utilized for decoration in the visited *terreiros*. It can be stated with frequency the use of the *búzios *from the species *M. moneta, M. annulus *e *E. caputserpentis *which are called *Ibás *(also known as "assentamentos," in the masks that decorate the orixás when they possess their sons to dance and in the guides of the bead necklaces (Figure 3).

The guides or the bead necklaces represent, through the determined colors that vary from Nation to Nation, the orixá owner of the *ori *of that son or daughter of saints. Bastide and Queiroz [42] considers the process of washing the necklaces as one of the rituals of the cycle of initiation in which the people that plan to follow Candomblé should submit themselves, constituted by the first step into the *terreiro *of the faithful, making the relationship with the orixá each time more profound. The guides (necklaces) offer protection to those that use it, impeding negative influence to interfere in the life of the adept. For these, besides the bath of determined herbs that vary in accordance to the *orixá *of each *filha/filho-de-santo*, it is necessary that the blood of the animals being offered in sacrifice is dropped over the necklace. This can be done at the time of the sacrificial ritual, where the guide will be put with the *Ibá *of the *orixá *being honored. Certain guides also represent the hierarchical degree of the *filho-de-santo*, *mocã *as an example, a braided straw necklace decorated with white shells (*M. annulus *or *M. moneta*) that have pending in its extremities two types of *vassourinhas *(brushes), that according to a priest, would symbolize the "sweeping away" of any poisonous influence that might harm the initiated (known as *Iaô*).

According with Santos [51], white colored búzios (*M. moneta *e *M. annulus*), alone, do not only symbolize the generic white, whose symbolism was previously discussed. The symbolize portions of this white, that is, units that resume or synthesize the interaction of two creating powers, the masculine and the feminine, in the right and the left side. Lacking the soft parts of the mollusks, the shells are still constituted of the symbol of the spiritual and ancestral *dobles *(one of the essential characteristics of the Nagô system would be that each spiritual or abstract element would correspond with a material or corporal representation or localization) [51].

**Figure 4:**
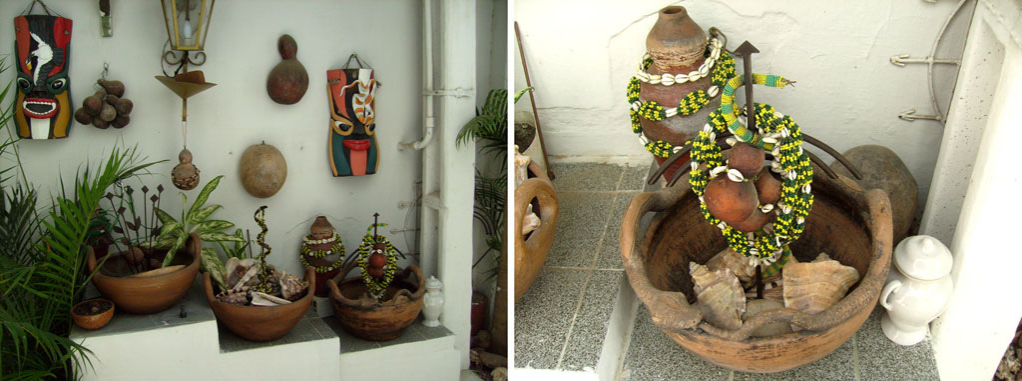
**On the left, *assentamentos *of the *orixás*, the two in the right representing Oxumarê and the one in the left end representing Ossãe**. On the right, details of the *Ibá *of Oxumarê, with *Cymatium raderi *and *Monetaria annulus *shells. Attempted representation of a serpent in the *Ibá*, since Oxumarê possess this symbology. From a *terreiro *of the Keto Nation in Caruaru (PE).

**Figure 5:**
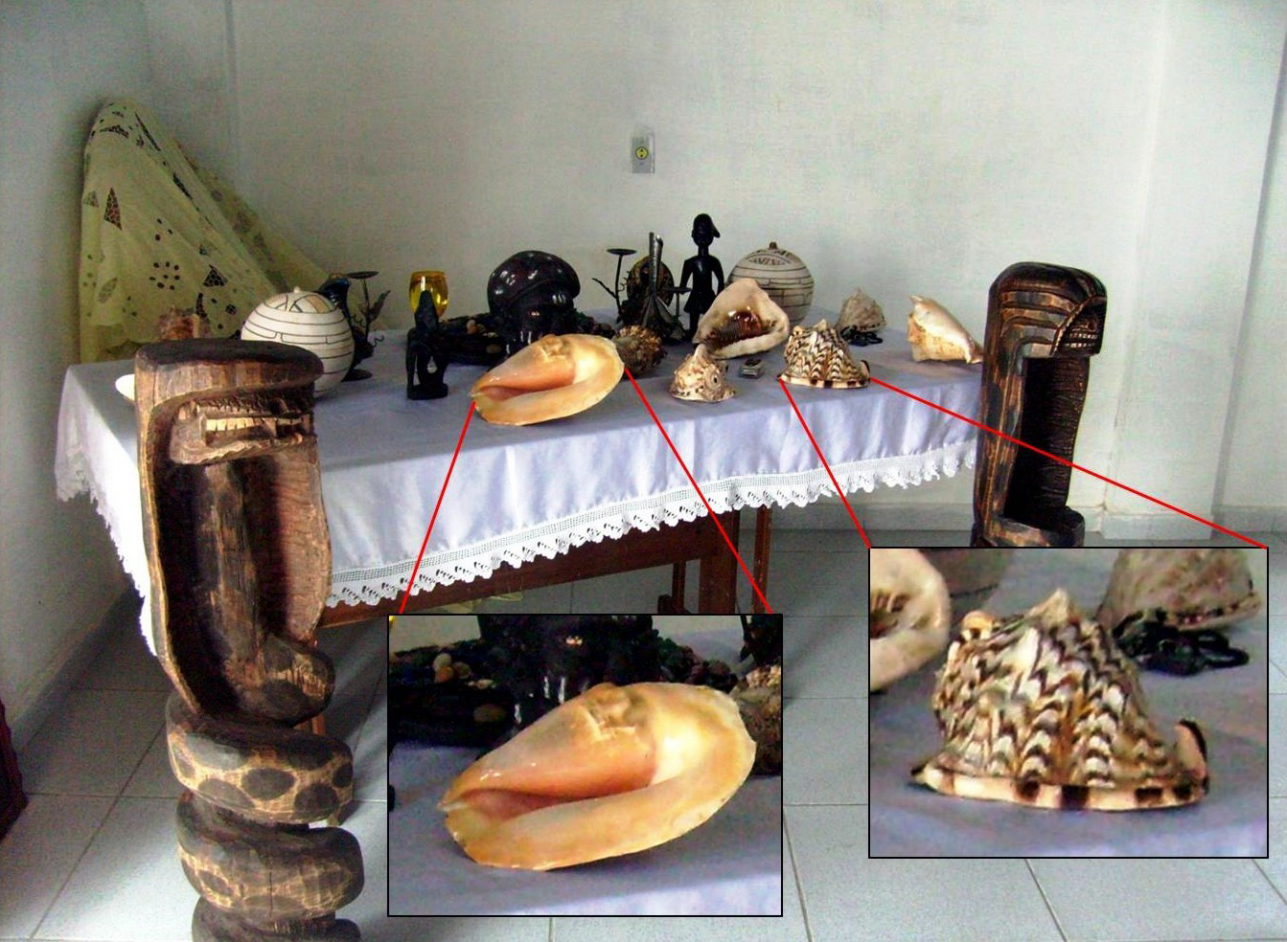
**Table on which the *babalorixá *consulted the "jogo de búzios," ornamented with shells of gastropods (*Eustrombus goliath *, *Cassis tuberosa (*in details), and *Charonia variegata*); the wooden sculptures of serpents symbolize the orixá Oxumarê**. In *terreiro *of the Yorubá Nation in Caruaru (PE).

The *Ibás *are constituted in specific locations for each supernatural entity, whose composing elements express the diverse aspects of the *orixá *whose nature is being symbolized [7,51]. Therefore, the *Ibás *possess the most diverse elements, varying from iron spears, *palhas da costa *(sacred fiber from West Africa), gourd, leaves, porcelain plates and small jars. According to Santos [51], in analyzing these elements and the structure of each *Ibá*, we could obtain precise materials for the research of the nature of the supernatural entities. This affirmation can be evidenced when, in visit to a *terreiro*, it was observed an *Ibá *of Oxumarê with the species of *Cassis tuberosa, Cymatium raderi *and *M. annulus *(Figure 4) in a table in which a *babalorixá *consulted the *búzios *where there existed diverse shells of the *C. tuberosa*, *E. goliath *and *Charonia variegata *decorating the same table (Figure 5).

When asked about the purpose of many shells in one *Ibá*, an interviewed *babalorixá *told the following myth:

Oxumarê was called to cure Olokum, the owner of the sea, father of Yemanjá. And Olokum was laying down, on the sand and dying, sick. And Oxumarê came and prepared a drink, with herbs, with everything and put in Olokum's mouth. Then Olokum was cured! When Olokum was cured, he stood up, and then Olokum ordered part of the riches of the sea and gave it to Oxumarê. Then Oxumarê rises to the skies and stays besides Ifá and the Orumilá. Then he gains those búzios, many sea shells and many pearls. Then he gains all of the wealth. Then Oxumarê is one of the very rich orixás that live beside Ifá and the Orumilá (Pai J. de Ogum).

From this myth, it is noted that the use of animals for ornamental means in Candomblé is not only constituted in the practice of decoration, but in a fundamental act in the religious teachings and in the mythic symbolism.

Silva [55], in the cities of Recife and Olinda, also registered the use of shells of the species *C. tuberosa *e *E. goliath *being used to ornament altars dedicated to the orixás.

The *búzio E. goliath *is being included in the National List of species of aquatic invertebrates and fishes endangered, overexploited or threatened of exploitation [72]. The registration of the same species associated with the religious use [55], suggests that the use of this mollusk for this finality is disseminated. New studies that can verify about the use of these mollusks in other *terreiros *of Candomblés, acting in conjunction with populational and ecological studies, become necessary to determine the magnitude and impact on the species. With is worth to emphasize that the use and the commerce of this species for ornamental means is common in various cities of Brazil, not only utilized by the adepts of Candomblé [11,12,73,74].

## The "White Blood"

As a sacrificial religion [8,42,43,51,54,56,66,67,70], Candomblé also utilizes mollusks for this religious finity. In this category, only the species *Achatina fulica *was cited. Known by the adepts of this religion as *Ibi, Igbin *or *"*Boi-de-Oxalá", this terrestrial gastropod is only offered to Oxalá. According to Santos [51], *igbin *represents one of the three blood categories, "the white blood." Every offering in honor to Oxalá must be of white color, due to the fact of it being included in the group of the *orixás-funfun*, of white. According to the symbolism, the *igbin *is equated to semen, which the *irunmalê *(supernatural entities, the orixás) possess due to their status as excellency, representing in this form the power of creation. The comparison of mollusks to the bull is done through the observation of the priest that when it is moving it directs its tentacles (called "antennas") forward, as if they are a bull's horn. As in the previous example, the *igbin *is the preferred offer of Oxalá.

**Figure 6:**
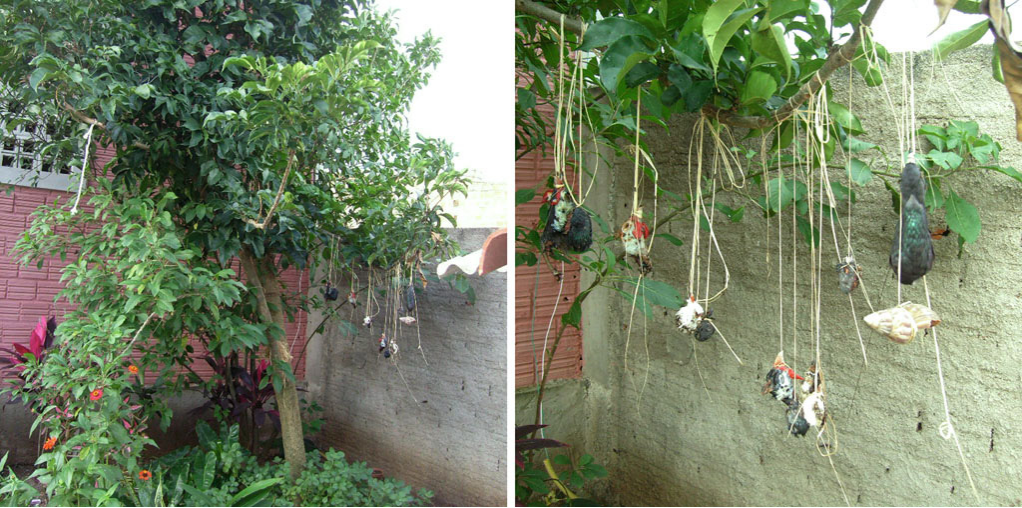
**In the left the tree in which the animal parts were hanging by straw strings**. In the right, details of the animals parts: to be cited the pigeon (*Columba livia*) and chicken (*galinhas-d'angola *- *Numida meleagris*) heads and a shell of *Achatina fulica*, called by the adepts the *Igbin*.

In one of the visited *terreiros*, it could be found a hanging shell of this mollusk, tied by a straw (*palha da costa*) string on a tree, together with pigeon (*Columbina livia*) and chicken heads (*Numida meleagris *- known as *conquém *by these adepts) (Figure 6). According to a *babalorixá*, these animal parts were drying in the sun, to later be grinded, making, together with other elements, a mixture that would be used to put on the head of the initiated (*Iaô)*. This mixture, called *Adoxu*, would symbolize the crest/hummock/of the *conquém*, a fact also observed by Vogel *et al.*[66], where it is highlighted the symbolic resemblance between the *Iaô *and the *conquém*.

## Final considerations

Nature and culture have a symbiotic relationship with traditional religion in many human societies [56]. In the Afro-Brazilian religious context, the mollusks assume an important function in Candomblé. Full of symbolisms that capacitate them, for example, in contacting the gods seeking the knowledge they wish, these animals become fundamental for the functioning of the cult, becoming sources of myth and enriching the Brazilian cultural patrimony.

Meanwhile, with all the extractive pressure, the capture of these mollusks, when in indiscriminate from, can bring risks to biodiversity. It is fit to highlight those conservational measures, if adopted, must be intimately connected to the social-cultural matters, since these animals are primordial elements to the religious cult, and as such they must be perceived. As pointed by Leo Neto *et al.*[56], given the undeniable influence that culture has on the way people perceive and use the resources of their environment, religion undoubtedly is indispensable to modern-day conservation and environmental protection efforts. Therefore, ethnozoological studies aimed at understanding human-animal relations from the perspective of religious traditions are essential in animal conservation.

## Competing interests

The authors declare that they have no competing interests.

## Authors' contributions

NALN, RAV, TLPD and RRNA - Writing of the manuscript, literature survey and interpretation; NALN, TLPD and RRNA- Ethnozoological data, and analysis of taxonomic aspects. All authors read and approved the final manuscript.
